# Effects of blood flow restriction therapy for lower limb dysfunction in stroke patients: a systematic review and meta-analysis

**DOI:** 10.3389/fneur.2026.1814592

**Published:** 2026-05-08

**Authors:** Jun Zhang, YuXin Xiao, LiangYu Qiu, Gang Zhang, Rui Wang, XuePing Zhu, Chan Wang, MingJun Xu

**Affiliations:** 1 Department of Acupuncture, Taihe Hospital, Hubei University of Medicine, Shiyan, Hubei, China; 2 Taihe Hospital, Hubei University of Medicine, Shiyan, Hubei, China; 3 Patient Follow-Up Center, Department of Nursing, Taihe Hospital, Hubei University of Medicine, Shiyan, Hubei, China

**Keywords:** blood flow restriction therapy, lower limb dysfunction, meta-analysis, motor function, stroke

## Abstract

**Objective:**

To systematically evaluate the efficacy of blood flow restriction training (BFRT) for lower limb dysfunction in stroke patients.

**Methods:**

Randomized controlled trials published up to October 2025 were retrieved from CNKI, Wanfang, VIP, SinoMed, PubMed, Embase, Web of Science, and Cochrane Library. The control group received conventional rehabilitation, while the intervention group received conventional rehabilitation combined with BFRT.

**Results:**

Eleven studies involving 522 patients were included. The intervention group achieved significantly higher Berg Balance Scale scores [MD = 4.52, 95% CI (0.78, 8.26), *p* = 0.02] and longer 6-min walk test distances [MD = 9.73, 95% CI (7.26, 12.21), *p* < 0.00001]. Subgroup analysis showed that when the intervention duration exceeded 4 weeks, the intervention group achieved higher lower-extremity Fugl-Meyer Assessment scores [MD = 4.38, 95% CI (2.00, 6.77), *p* = 0.0003] and Modified Barthel Index scores [MD = 10.50, 95% CI (6.32, 14.68), *p* < 0.00001] than the control group. Among patients in the subacute phase, the intervention group also demonstrated higher lower-extremity Fugl-Meyer Assessment scores [MD = 2.63, 95% CI (0.42, 4.83), *p* = 0.02].

**Conclusion:**

BFRT improves balance and walking endurance in stroke patients. When delivered for more than 4 weeks, it yields additional benefits in lower limb motor function and activities of daily living, with more pronounced motor improvements observed in the subacute phase. Owing to limitations in sample size, treatment protocol heterogeneity, and methodological quality, these findings require confirmation through large-scale, high-quality studies.

## 1 Introduction

Stroke is one of the leading causes of long-term disability worldwide. According to the World Stroke Report 2025, the global number of stroke survivors has exceeded 1.001 billion, with more than half experiencing persistent motor impairments due to hemiplegia, severely impacting their quality of life (1, 2). Lower limb dysfunction, one of the most common complications following a stroke, is characterized by impaired balance control (affecting 83% of survivors) (3), reduced walking ability (only 37% of patients can walk independently) (4), and increased dependence in activities of daily living (ADL) (approximately 30–40% of patients experience a long-term loss of functional ambulation) (5, 6). Consequently, improving lower limb motor function, enhancing balance, and increasing walking capacity have become core objectives of stroke rehabilitation, representing critical clinical challenges that demand urgent attention (2).

In recent years, blood flow restriction training (BFRT) has emerged as a novel rehabilitation technique and has been progressively integrated into neurological rehabilitation. This technique involves applying adjustable external pressure to the proximal portion of the limb in conjunction with low-intensity exercise (20–30% of one-repetition maximum). This creates a local ischemic-reperfusion environment, which triggers a metabolic stress response, leading to increased recruitment of fast-twitch muscle fibers, enhanced muscle protein synthesis, and improved neuromuscular adaptation (7, 8). Owing to its low exercise intensity and favorable safety profile, BFRT is particularly suitable for stroke survivors, who often present with muscle weakness and low fatigue tolerance (9). BFRT has demonstrated significant efficacy in sports medicine and orthopedic rehabilitation (10, 11). Preliminary clinical studies suggest that it can effectively improve muscle mass, enhance muscle activation, and positively influence certain functional outcomes in stroke patients (12, 13). However, research on the application of BFRT in lower limb stroke rehabilitation remains limited. Most existing studies are single-center trials with small sample sizes, and the training protocols lack standardization (e.g., inconsistencies in occlusion pressure, cuff placement, and training frequency). Furthermore, most studies have focused on muscle strength or isolated functional measures, leading to inconclusive or even controversial findings (14). Therefore, this meta-analysis was conducted to comprehensively evaluate the efficacy of BFRT on lower limb dysfunction in stroke patients across four key domains: lower limb motor function, balance, walking ability, and activities of daily living. Furthermore, a subgroup analysis was performed to investigate the influence of intervention duration on treatment outcomes, aiming to provide more robust evidence for the clinical application of BFRT.

## 2 Data methods

This systematic review and meta-analysis was conducted in accordance with the Cochrane Handbook for Systematic Reviews of Interventions. The review protocol was prospectively registered with the PROSPERO database (National Institute for Health Research, UK) under the registration number [CRD42025108622].

### 2.1 Inclusion criteria

The inclusion criteria were as follows: (1) Types of studies: randomized controlled trials (RCTs), the inclusion language was limited to Chinese and English, with no restriction on age, gender, race, or ethnicity; (2) Types of participants: patients diagnosed with stroke (15) (confirmed by CT or MRI), aged >18 years, with lower limb dysfunction; (3) Types of interventions: the control group received conventional rehabilitation (e.g., physical therapy, occupational therapy, neuromodulation techniques, and traditional rehabilitation), while the experimental group received BFRT in addition to conventional rehabilitation; (4) Types of outcome measures: lower limb motor function, balance function, walking ability, and ADL.

### 2.2 Exclusion criteria

Studies were excluded if they: (1) did not meet the prespecified inclusion criteria; (2) were duplicate publications; (3) had incomplete data that could not be obtained after contacting the corresponding authors; (4) had full texts that could not be accessed; (5) were reviews, case reports, conference abstracts, or animal studies.

### 2.3 Database and retrieval strategy

A systematic search was conducted in the following databases from their inception to October 2025: China National Knowledge Infrastructure, Wan Fang Database, Chinese Science and Technology Periodical Databases, SinoMed, PubMed, Embase, Web of Science, and the Cochrane Library. The search aimed to identify RCTs investigating the effects of BFRT on lower limb dysfunction in stroke patients. A combination of subject headings and free-text terms was used, and the reference lists of the included studies were manually screened. The complete search strategy employed for the PubMed database is presented in Table 1.

**Table 1:** Search strategy for the PubMed database.

Number	Search term
#5	#3 AND #4
#4	(Blood Flow Restriction Therapy [Mesh Terms) OR (Blood Flow Restriction Training [Title/Abstract]) OR (Blood Flow Restriction Exercise [Title/Abstract]) OR (BFR Therapy [Title/Abstract])) OR (Therapy, BFR[Title/Abstract])
#3	#1 OR #2
#2	(Strokes[Title/Abstract])) OR (Cerebrovascular Accident [Title/Abstract])) OR (Cerebral Stroke [Title/Abstract])) OR (Stroke, Cerebral[Title/Abstract])) OR (Cerebrovascular Apoplexy[Title/Abstract])) OR (Brain Vascular Accident [Title/Abstract])) OR (Cerebrovascular Stroke[Title/Abstract])) OR (Apoplexy[Title/Abstract])) OR (CVA [Title/Abstract])) OR (Acute Stroke [Title/Abstract])) OR (Acute Cerebrovascular Accident [Title/Abstract])) OR (Hemorrhagic Stroke[Title/Abstract]) OR (Ischemic Stroke[Title/Abstract])
#1	Stroke [MeSH Terms]

### 2.4 Outcome measures

#### 2.4.1 Primary outcome

Balance function was assessed using the Berg Balance Scale (BBS), which consists of 14 items, each scored from 0 to 4, for a maximum total score of 56. Higher scores indicate better balance ability.

#### 2.4.2 Secondary outcomes

(1) Lower limb motor function was evaluated using the Fugl-Meyer Assessment for Lower Extremity (FMA-LE). This scale comprises 17 items, each rated from 0 to 2, yielding a maximum score of 34, with higher scores reflecting better motor function. (2) Walking ability was measured using the 6-Minute Walk Test (6MWT), which assesses walking endurance, and the Timed Up and Go Test (TUGT), which evaluates functional mobility. (3)ADL were assessed using the Modified Barthel Index (MBI), which consists of 10 items with a maximum total score of 100; higher scores indicate greater independence in ADL.

### 2.5 Data collection and extraction

Two researchers independently screened the literature, extracted data, and cross-checked the extracted data against the inclusion and exclusion criteria. Any disagreements were resolved through discussion or by consulting a third researcher. The following information was extracted from each included study: first author, publication year, sample size, participant characteristics (age, sex, disease duration), intervention details (type, protocol, and duration), and outcome measures.

### 2.6 Risk of bias assessment

The methodological quality of the included studies was assessed using the Cochrane Risk of Bias Assessment Tool (16). This tool evaluates seven domains: random sequence generation, allocation concealment, blinding of participants and personnel, blinding of outcome assessment, incomplete outcome data, selective reporting, and other sources of bias. Each domain was rated as having a “low risk of bias,” “high risk of bias,” or “unclear risk of bias.”

### 2.7 Statistical analysis

A meta-analysis was performed using RevMan 5.4 software. For continuous outcomes, the mean difference (MD) or standardized mean difference (SMD) with 95% confidence intervals (CIs) was calculated as the effect measure. Statistical heterogeneity was assessed using the *I*^2^ statistic and the *Q* test (*p*-value). A fixed-effects model was applied when *p* > 0.1 and *I*^2^ < 50%; otherwise, a random-effects model was used when *p* ≤ 0.1 and *I*^2^ ≥ 50%. For outcomes with significant heterogeneity, sensitivity analyses or subgroup analyses were conducted to explore potential sources of heterogeneity. Based on sources of clinical heterogeneity, this study planned to conduct the following subgroup analyses: (1) intervention duration (≤4 weeks vs. >4 weeks); (2) disease duration was classified as acute phase (≤1 month), subacute phase (1–6 months), and chronic phase (>6 months); and (3) intervention modality (isolated BFRT vs. combined multimodal intervention). Among these, the subgroup analysis by intervention modality could not be performed due to the insufficient number of studies in the isolated blood flow restriction training group (only 1 study); therefore, only a descriptive analysis was conducted.

## 3 Results

### 3.1 Literature screening results

**Figure 1:**
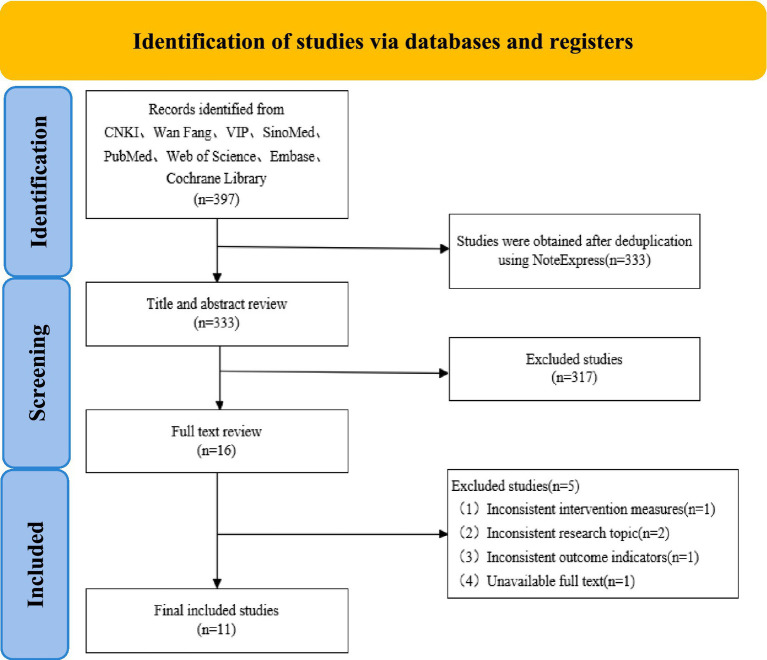
Literature screening flowchart.

A total of 397 records were identified through database searching. After removing duplicates (*n* = 64), 333 records were screened based on titles and abstracts, of which 317 were excluded. The remaining 16 full-text articles were assessed for eligibility, and 5 were excluded with reasons. Finally, 11 studies were included in the qualitative and quantitative synthesis (meta-analysis). The literature screening process is illustrated in Figure 1.

### 3.2 Characteristics of included studies

A total of 11 studies published between 2021 and 2025 (17–27) were included, comprising 522 patients, with 261 in the experimental group and 261 in the control group. The sample included 335 males and 187 females, all aged over 40 years. The intervention duration ranged from 2 to 8 weeks. The basic characteristics of the included studies are summarized in Table 2.

**Table 2:** Basic characteristics of the included studies.

Study	Sample T/C	Sex (male/female)T/C	Age (Years) T/C	Clinical course T/C
Yang (17) 2021	33/33	T:15/18:14/19	T:46.21 ± 4.98:46.26 ± 5.21	T:17.95 ± 3.52d C:18.23 ± 3.62d
Du et al. (18) 2022	30/30	T:18/12:17/13	T:66.8 ± 11.2:67.2 ± 10.8	T:76.4 ± 18.4d C:75.6 ± 11.1d
Du et al. (19) 2022	30/30	T:17/13:18/12	T:51.1 ± 3.9:51.8 ± 4.2	—
Feng et al. (20) 2023	15/14	T:10/5:11/3	T:54.07 ± 10.44:45.29 ± 13.74	T:12.27 ± 11.55m C:14.36 ± 15.45m
Xu et al. (21) 2023	29/29	T:19/10:24/5	T:60.10 ± 11.22:59.41 ± 11.85	T:1.58 ± 1.28m C:1.60 ± 1.27m
Sun et al. (22) 2024	27/28	T:15/12:18/10	T:70.70 ± 4.91:69.93 ± 5.89	T:42.63 ± 21.48d C:51.75 ± 26.56d
Ahmed et al. (23) 2024	15/15	T:10/5:9/6	T:54.20 ± 13.19:54.73 ± 14.08	T:25.60 ± 29.89m C:23.06 ± 31.45m
Gong et al. (24) 2025	26/26	T:17/9:13/13	T:57.5 ± 12.17: 61.69 ± 9.10	T:27.42 ± 6.73d C:26.19 ± 4.6d
Tang et al. (25) 2025	16/16	T:16/4:14/6	T:52 ± 12:52 ± 9	T:32 ± 12d C:27 ± 14d
Shang et al. (26) 2025	20/20	T:16/4:14/6	T:54.40 ± 6.86:54.27 ± 7.18	T:8.67 ± 1.72wk C:8.20 ± 1.72wk
Feng et al. (27) 2025	20/20	T:15/5:15/5	T:52.70 ± 11.12:44.85 ± 12.99	T:13.50 ± 14.46m C:13.13 ± 15.20m

### 3.3 Quality of evidence

**Figure 2:**
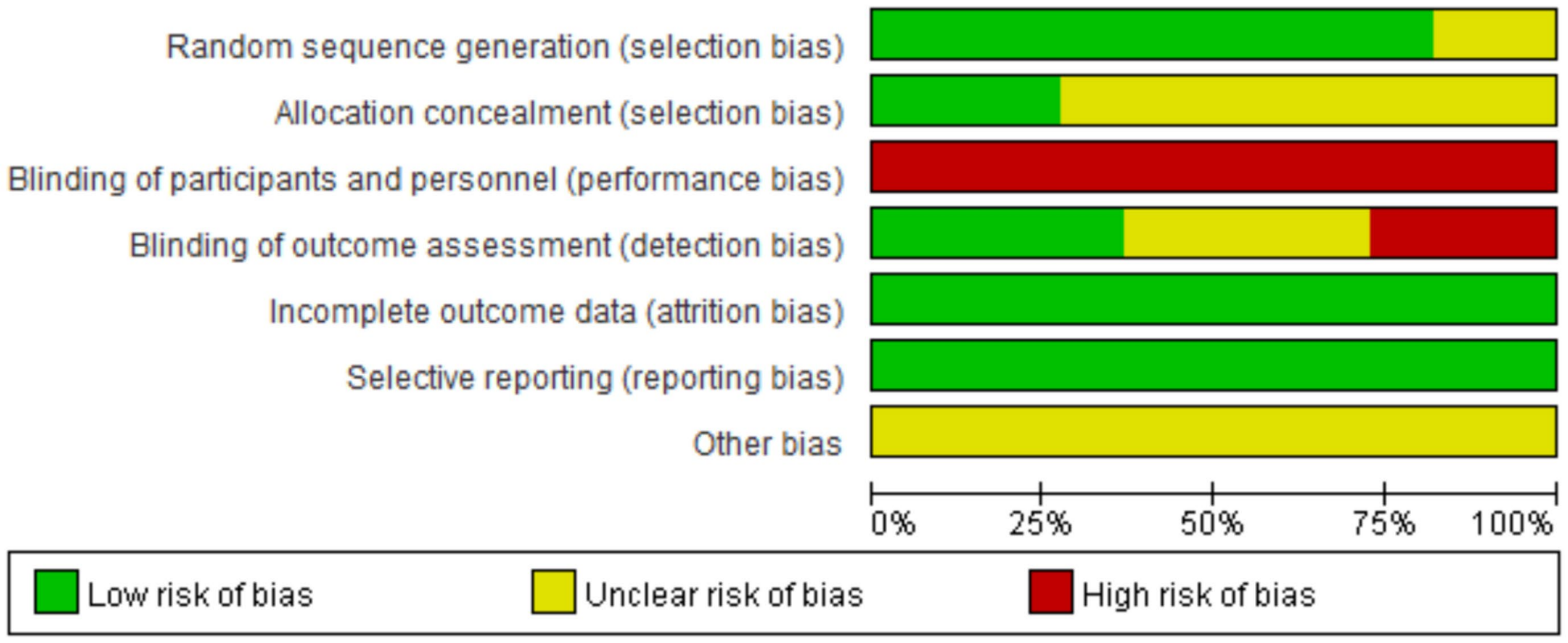
Risk of bias graph.

**Figure 3:**
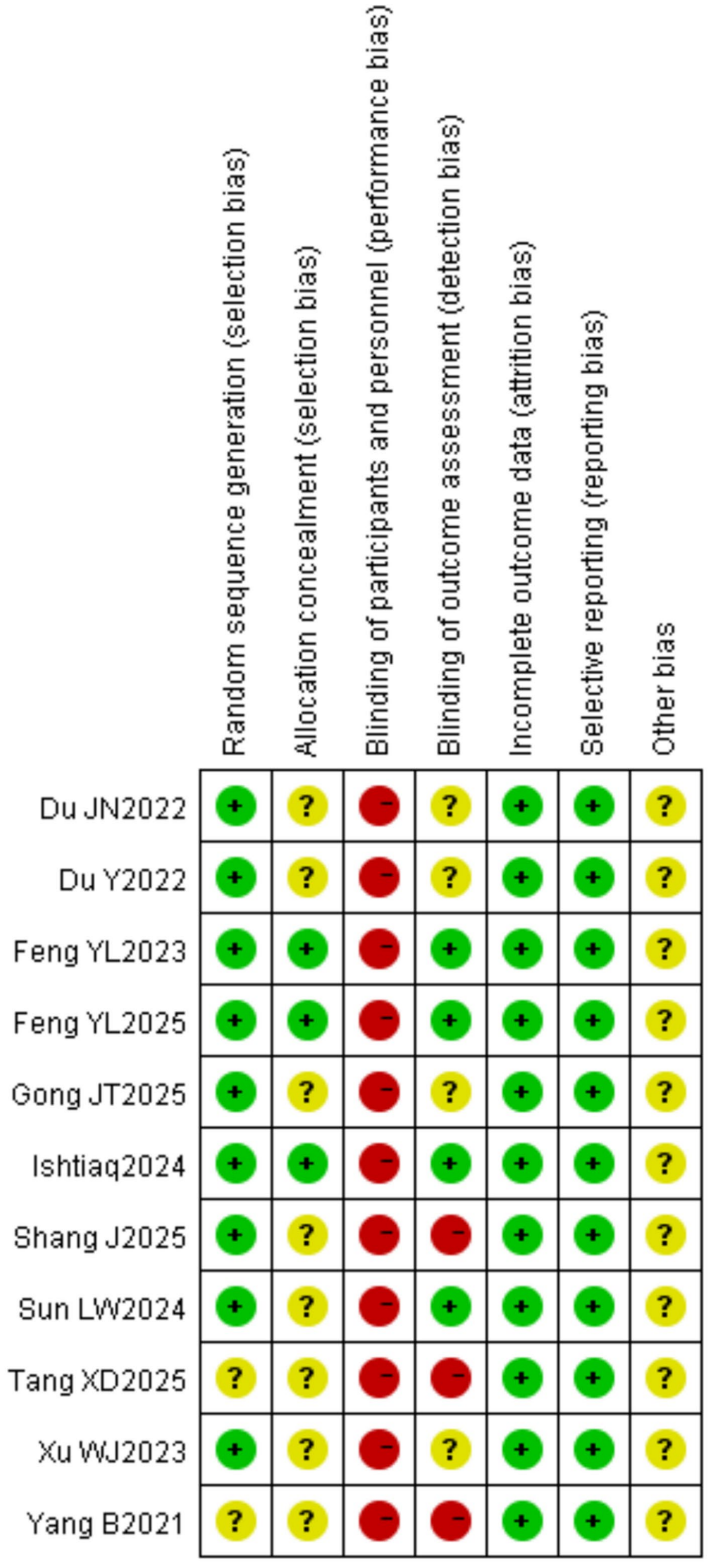
Risk of bias summary.

The methodological quality of the included studies was assessed using the Cochrane Risk of Bias Tool. All 11 studies reported random assignment (17–27); of these, nine specified the method of random sequence generation (random number table) (18–24, 26, 27), while two only stated “random” without providing details (17, 25). Allocation concealment was implemented in three studies (20, 23, 27). Regarding blinding, none of the 11 studies reported blinding of participants or personnel (17–27); however, four studies blinded outcome assessors (20, 22, 23, 27). No incomplete outcome data or selective reporting were identified in any of the studies, while other sources of bias were unclear. Based on the overall quality assessment, nine studies were rated as grade B (18–24, 26, 27) and two as grade C (17, 25). The risk of bias summary is presented in Figures 2, 3.

### 3.4 Meta-analysis results

#### 3.4.1 BBS

**Figure 4:**
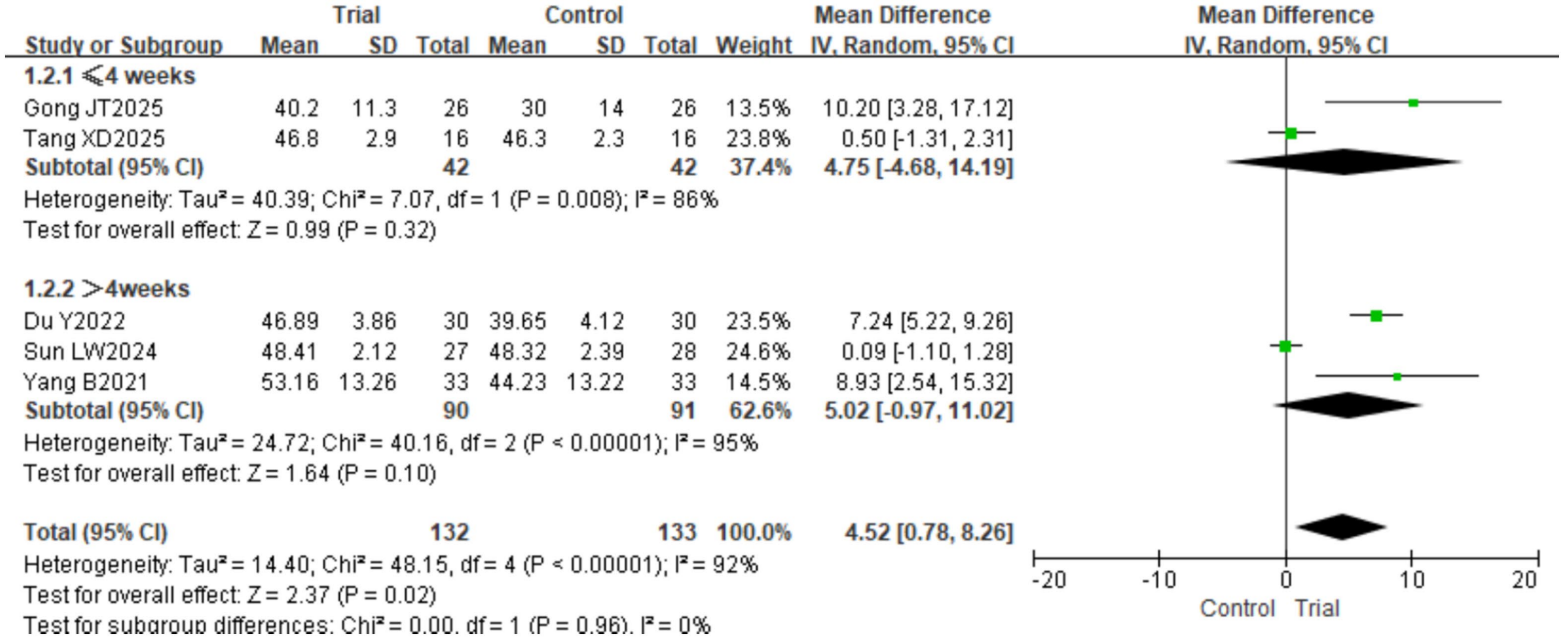
Forest plot of BBS scores by subgroup analysis based on intervention duration.

**Figure 5:**
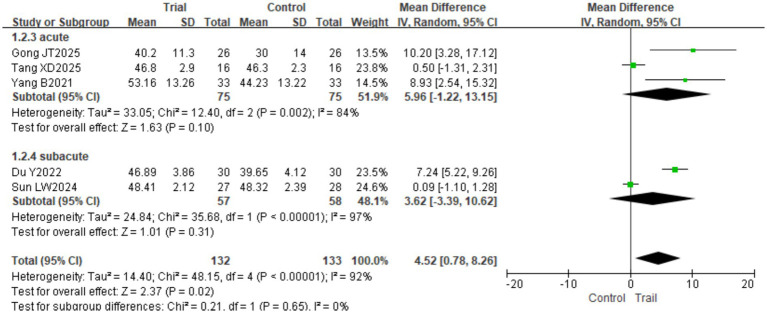
Forest plot of BBS scores by subgroup analysis based on disease duration.

A total of 5 studies (17, 18, 22, 24, 25) involving 265 patients (132 in the intervention group and 133 in the control group) were included. Significant heterogeneity was detected among the studies (*I*^2^ = 92%, *p* < 0.00001); therefore, a random-effects model was employed. The pooled results demonstrated that the intervention group achieved significantly higher BBS scores than the control group [MD = 4.52, 95% CI (0.78, 8.26), *p* = 0.02]. Subgroup analysis based on intervention duration (≤4 weeks vs. >4 weeks) revealed no statistically significant between-group differences (*p* > 0.05). The results are presented in Figure 4. Based on disease duration, patients were classified into the acute phase (≤1 month), subacute phase (1–6 months), and chronic phase (>6 months). As Du JN’s original study did not report specific duration data, we reviewed the original information and classified the patients as being in the subacute phase for analysis. Subgroup analyses based on disease duration revealed no statistically significant differences between groups in either the acute or subacute phases (*p* > 0.05). The results are presented in Figure 5.

#### 3.4.2 FMA-LE

**Figure 6:**
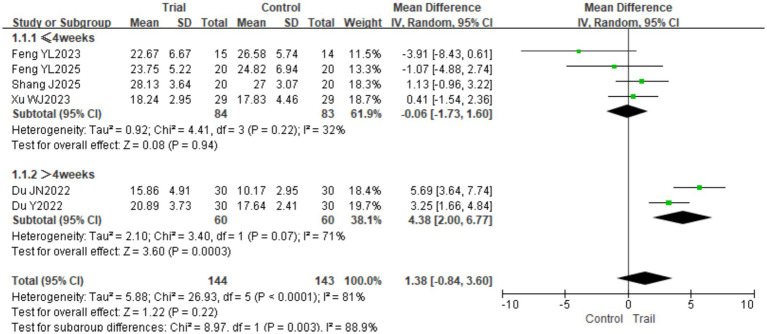
Forest plot of FMA-LE scores by subgroup analysis based on intervention duration.

**Figure 7:**
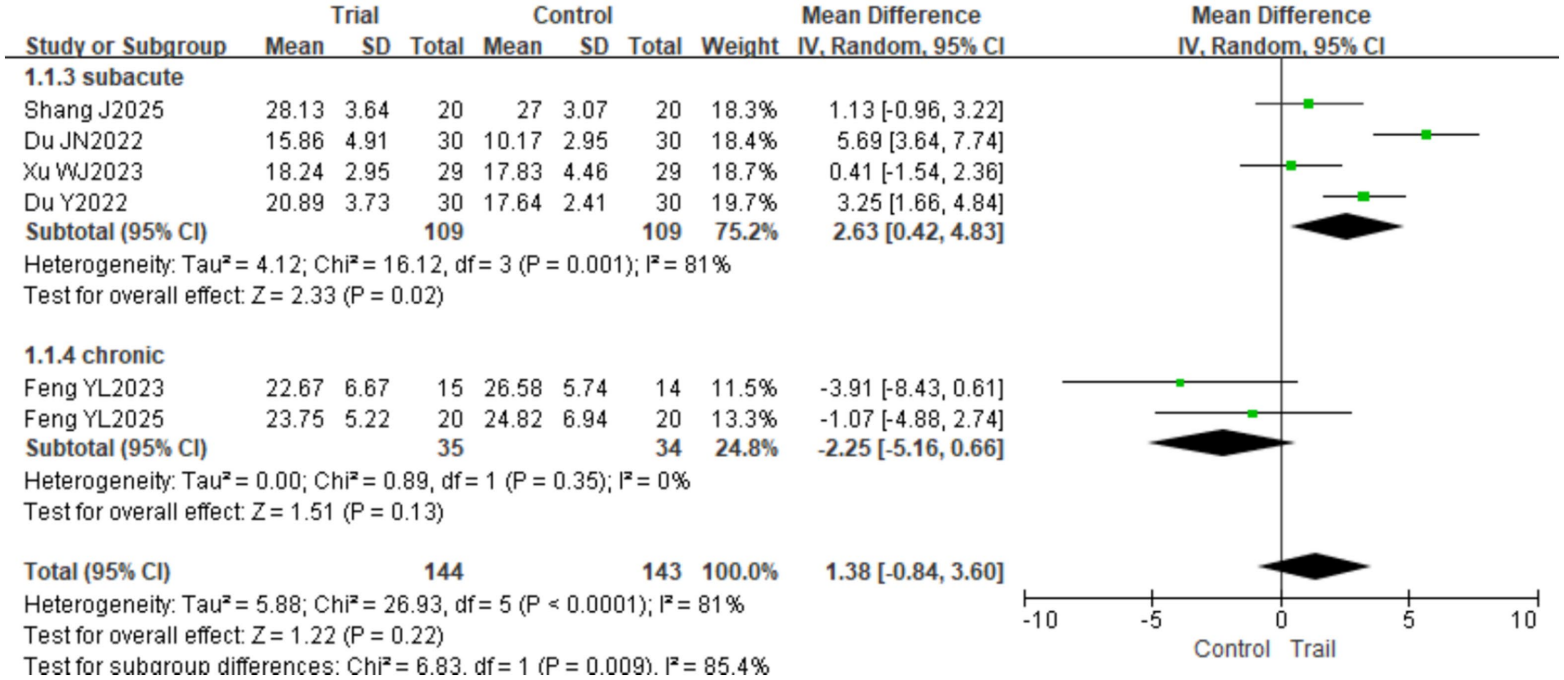
Forest plot of FMA-LE scores by subgroup analysis based on disease duration.

A total of six studies (18–21, 26, 27) involving 287 patients (144 in the experimental group and 143 in the control group) were included. Significant heterogeneity was detected among the studies (*I*^2^ = 81%, *p* < 0.0001); therefore, a random-effects model was employed. The pooled analysis showed no significant difference in FMA-LE scores between the two groups [MD = 1.38, 95% CI (−0.84, 3.60), *p* = 0.22]. Subgroup analysis based on intervention duration revealed that when the intervention lasted ≤4 weeks, there was no significant difference between groups [MD = −0.06, 95% CI (−1.73, 1.60), *p* = 0.94]; however, when the intervention duration exceeded 4 weeks, the experimental group achieved significantly higher FMA-LE scores than the control group [MD = 4.38, 95% CI (2.00, 6.77), *p* = 0.0003]. The results are presented in Figure 6. Subgroup analysis based on disease duration revealed the following results: in the subacute phase, the experimental group had significantly higher scores on the FMA-LE than the control group [MD = 2.63, 95% CI (0.42, 4.83), *p* = 0.02]; in the chronic phase, there was no significant difference between the two groups [MD = −2.25, 95% CI (−5.16, 0.66), *p* = 0.13]. The results are presented in Figure 7.

#### 3.4.3 6MWT

**Figure 8:**
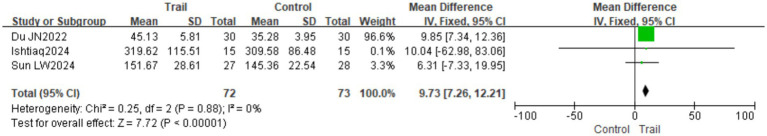
Forest plot of 6MWT.

A total of three studies (19, 22, 23) involving 145 patients (72 in the experimental group and 73 in the control group) were included. No significant heterogeneity was detected among the studies (*I*^2^ = 0%, *p* = 0.88); therefore, a fixed-effects model was employed. The pooled results showed that the 6MWT distance was significantly greater in the experimental group than in the control group [MD = 9.73, 95% CI (7.26, 12.21), *p* < 0.00001]. The results are presented in Figure 8.

#### 3.4.4 TUGT

**Figure 9:**
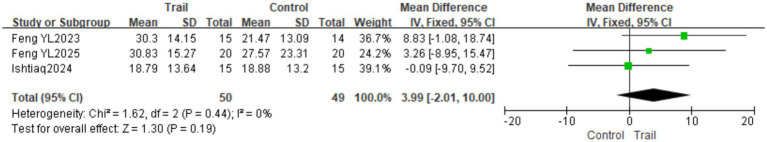
Forest plot of TUGT.

A total of three studies (20, 23, 27) involving 99 patients (50 in the experimental group and 49 in the control group) were included. No significant heterogeneity was detected among the studies (*I*^2^ = 0%, *p* = 0.44); therefore, a fixed-effects model was employed. The pooled results showed no significant difference in TUGT times between the two groups [MD = 3.99, 95% CI (−2.01, 10.00), *p* = 0.19]. The results are presented in Figure 9.

#### 3.4.5 MBI

**Figure 10:**
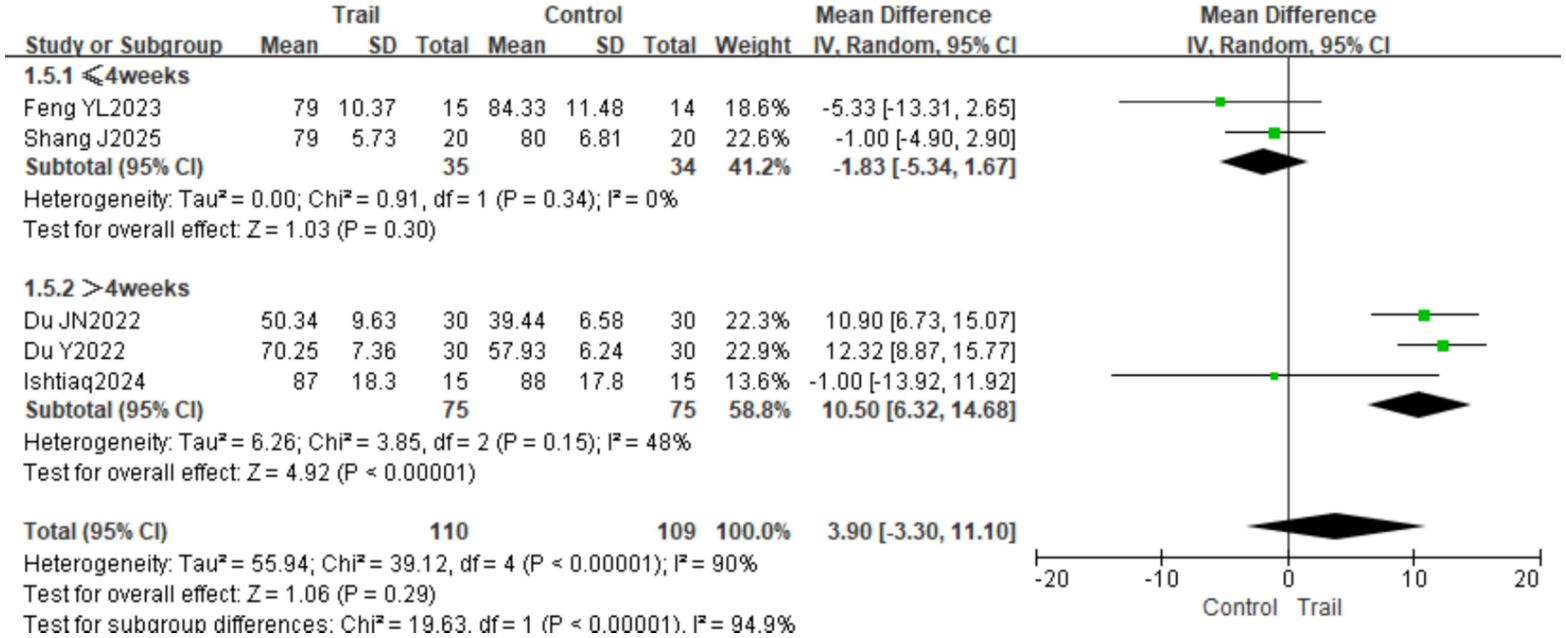
Forest plot of MBI scores by subgroup analysis based on intervention duration.

**Figure 11:**
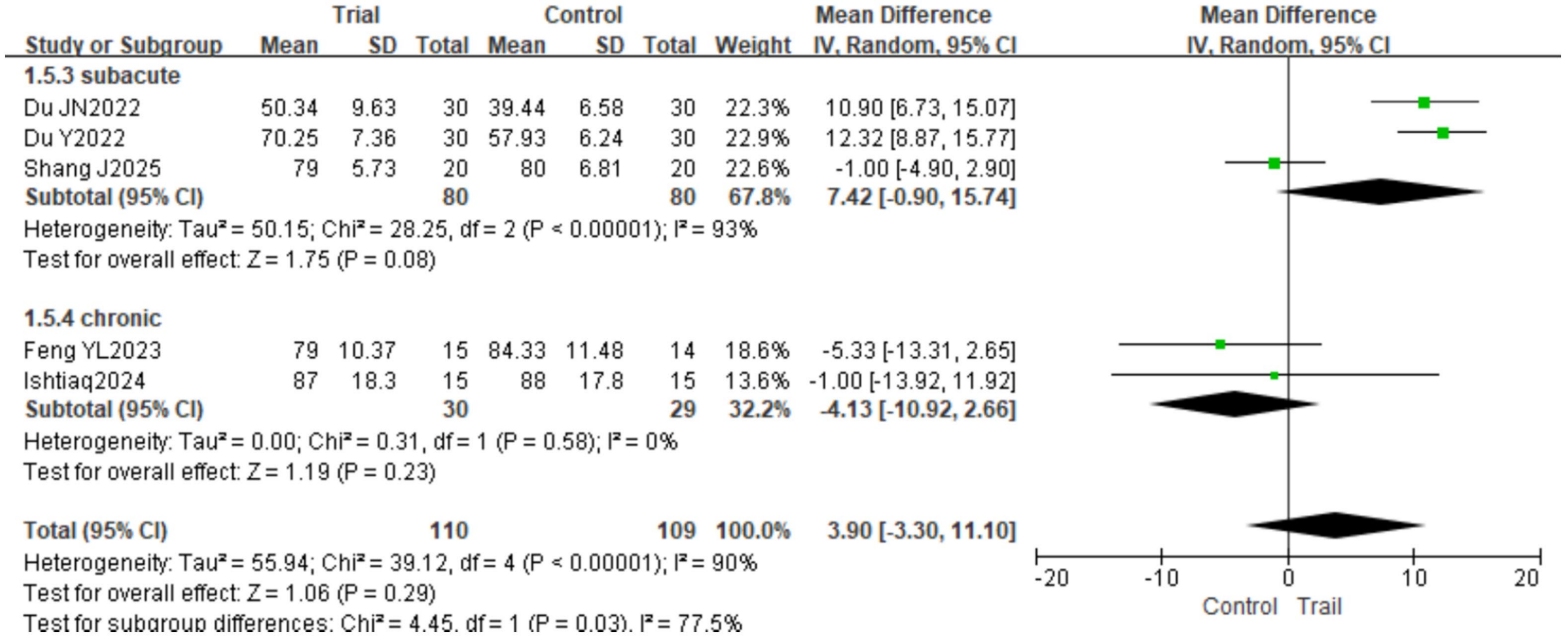
Forest plot of MBI scores by subgroup analysis based on disease duration.

A total of five studies (18–20, 23, 26) involving 219 patients (110 in the intervention group and 109 in the control group) were included. Significant heterogeneity was detected among the studies (*I*^2^ = 90%, *p* < 0.00001); therefore, a random-effects model was employed. The pooled analysis showed no significant difference in MBI scores between the two groups [MD = 3.90, 95% CI (−3.30, 11.10), *p* = 0.29]. Subgroup analysis based on intervention duration revealed that when the intervention lasted ≤4 weeks, there was no significant difference in MBI scores between the groups [MD = −1.83, 95% CI (−5.34, 1.67), *p* = 0.30]; however, when the intervention duration exceeded 4 weeks, the intervention group achieved significantly higher MBI scores than the control group [MD = 10.50, 95% CI (6.32, 14.68), *p* < 0.00001]. The results are presented in Figure 10. Subgroup analysis based on disease duration revealed no statistically significant differences between groups in either the acute or subacute phases (*p* > 0.05). The results are presented in Figure 11.

#### 3.4.6 Analysis of intervention modes

An analysis of the intervention modes in the included studies revealed that only one study (25) involved isolated BFRT (intervention group: BFRT + conventional rehabilitation; control group: conventional rehabilitation). The remaining 10 studies (17–24, 26, 27) all employed combined multimodal interventions, in which the experimental group received BFRT in addition to a specific active intervention (e.g., acupuncture, resistance training, vibration training, transcranial magnetic stimulation, suspension training), while the control group received the same active intervention without BFRT. The current evidence primarily reflects the efficacy of BFRT when used as an adjunct to other active interventions (see Table 3).

**Table 3:** Basic characteristics of the included studies.

Study	Intervention T/C	Material composition	Location	Compression (mmHg)	Treatment duration (week)	Outcome measures
Yang (17) 2021	T: BFRT + resistance training C: resistance training	Nylon pressure band and sphygmomanometer	Midshaft of the femur	60–170	6	①②
Du et al. (18) 2022	T: BFRT + resistance training C: resistance training	Compression cuff	Midshaft of the femur	140–200	8	①②⑤
Du et al. (19) 2022	T: BFRT + rTMS + Conventional rehabilitation C: rTMS + Conventional rehabilitation	Bstrong Training Bands (USA)	Midshaft of the femur	250	6	①③⑤
Feng et al. (20) 2023	T: BFRT + exercise training C: Exercise Training	Bstrong Training Bands (USA)	Proximal thigh	160–200	3	①④⑤
Xu et al. (21) 2023	T: BFRT + resistance training C: resistance training	Tourniquet	Mid-thigh	200	2	①
Sun et al. (22) 2024	T: BFRT + vibration training C: Vibration training	Compression Cuff	Upper thigh	140–200	6	②③
Ahmedet al (23). 2024	T: BFRT + resistance training C: resistance training	Standard blood pressure cuff	Proximal thigh	150–160	5	③④⑤
Gong et al. (24) 2025	T: BFRT + Scalp acupuncture + Conventional rehabilitation C: Conventional rehabilitation +Scalp acupuncture	Non-elastic training bands (Theratool brand)	Mid-thigh	100–240	4	②
Tang et al. (25) 2025	T: BFRT + Conventional rehabilitation C: Conventional rehabilitation	Inflatable cuff	Proximal thigh	70	2	②
Shang et al. (26) 2025	T: BFRT + Uspension Training +Conventional rehabilitation C: Conventional rehabilitation + Uspension training	Tbfr occlusion training bands	Proximal thigh	200	4	①⑤
Feng et al. (27) 2025	T: BFRT + exercise training C: exercise training	Bstrong training bands (USA)	Proximal thigh	160–200	4	①④

*T* is the test group, C is the control group, “–” means no data. ①Fugl-Meyer Lower Extremity Motor Function Scale; ② Berg Balance Scale; ③6-min walk test; ④Time Up and Go Test; ⑤ Modified Barthel Index; ⑥repetitive Transcranial Magnetic Stimulation.

### 3.5 Sensitivity analysis

After sequentially excluding studies based on the FMA-LE and MBI metrics, the results did not change significantly, indicating robust findings.

**Figure 12:**
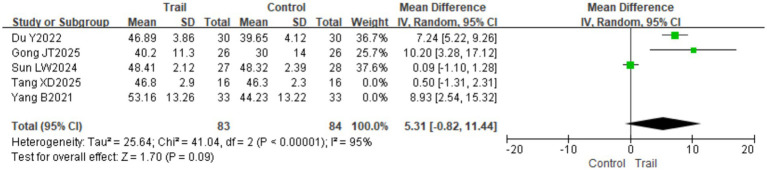
Forest plot of BBS scores after excluding high-risk studies.

For the BBS index, after excluding two high-risk studies (17, 25), the pooled effect was no longer statistically significant [MD = 5.31, 95% CI (−0.82, 11.44), *p* = 0.09]. This change may be attributed to the following factors. First, the study by Yang et al. (17) had a large sample size (66 cases) and thus carried considerable weight in the meta-analysis; it also included a specific population of obese stroke patients who may have exhibited a stronger response to the intervention. The study by Tang et al. (25) included patients in the acute phase (within 8 weeks of onset), who generally have high rehabilitation potential, but the intervention duration was short (2 weeks), and its short-term effects may have amplified the overall effect size. Additionally, according to the Cochrane Risk of Bias Assessment Tool, both studies had potential risks of bias, such as unclear randomization methods and lack of blinding, which may have led to overestimation of the intervention effects. After excluding these two studies, only three studies remained; the reduced sample size and consequent decrease in statistical power resulted in the loss of statistical significance. These findings suggest that the robustness of the conclusions requires further validation through additional high-quality, large-sample RCTs. The results are presented in Figure 12.

### 3.6 Publication bias

Publication bias was not assessed using funnel plots, as fewer than 10 studies were included in the meta-analysis, which is below the recommended threshold for this method (28). Therefore, only a descriptive assessment of publication bias was conducted. Given that the included RCTs had relatively small sample sizes, a certain risk of publication bias may exist.

## 4 Discussion

Lower limb dysfunction following stroke represents a key focus and challenge in comprehensive rehabilitation. This systematic review and meta-analysis evaluated the effects of BFRT on lower limb motor function, balance, walking ability, and ADL in stroke patients. A total of 11 RCTs involving 522 patients were included. The pooled results demonstrated that BFRT significantly improved balance function. Moreover, long-term intervention (duration > 4 weeks) further enhanced recovery of lower limb motor function, walking endurance, and ADL, revealing a clear time-dependent cumulative effect. However, no significant improvement was observed in walking agility, as measured by the TUGT.

The overall pooled estimates for the FMA-LE and the MBI showed no significant differences between groups (*p* > 0.05). However, subgroup analysis based on intervention duration revealed a significant “time-effect” relationship: when intervention exceeded 4 weeks, BFRT significantly improved both FMA-LE scores (MD = 4.38, *p* = 0.0003) and MBI scores (MD = 10.50, *p* < 0.00001). This suggests that the therapeutic efficacy of BFRT in stroke patients exhibits pronounced time-dependence and cumulative dose effects. The FMA-LE assesses motor function across multiple dimensions, including reflex activity, synergistic movement, and coordination control. Improvement in motor function depends not only on increased muscle strength but also on central nervous system (CNS) remodeling and the relearning of movement patterns (28). When intervention duration is ≤ 4 weeks, muscle strength gains may result primarily from localized metabolic stress and muscle fiber recruitment, which may be insufficient to trigger extensive neural adaptive changes. In contrast, with intervention exceeding 4 weeks, the CNS has sufficient time for remodeling, enabling reconstruction of synergistic patterns between the motor cortex and spinal segments, thereby leading to marked functional improvements. Similarly, recovery of ADL (e.g., eating, dressing, walking) builds upon multidimensional improvements in lower limb motor function, gait, and balance. As a higher-order integrated function, improvement in the Barthel Index relies on prolonged, adequately dosed intervention stimulation (29). Motor learning theory and principles of functional reconstruction indicate that functional recovery requires sustained, high-intensity, high-repetition task-oriented training over a specific period to accumulate training effects. These findings suggest that future studies should pay particular attention to the impact of intervention duration on efficacy, with patients deriving greater benefit when intervention exceeds 4 weeks.

In addition to intervention duration, disease stage also influenced treatment efficacy. Subgroup analysis showed that among patients in the subacute phase (1–6 months), the BFRT group had significantly higher FMA-LE scores than the control group [MD = 2.63, 95% CI (0.42, 4.83), *p* = 0.02], whereas no significant difference was observed in the chronic phase (>6 months). This suggests that initiating BFRT during the subacute phase may be more beneficial for improving lower limb motor function. Potential mechanisms include enhanced neuroplasticity, less established muscle atrophy, and greater integrity of the residual corticospinal tract during this stage, all of which may facilitate a better treatment response (30). Taken together, these findings highlight the importance of considering disease stage in clinical practice, with earlier intervention in the subacute phase potentially offering greater therapeutic advantages.

Regarding balance function, the pooled analysis demonstrated a significant improvement in BBS scores favoring the BFRT group (MD = 4.52, *p* = 0.02), indicating that BFRT effectively enhances balance in stroke patients. However, subgroup analysis based on intervention duration revealed no significant between-group differences, regardless of whether the duration was ≤4 weeks or >4 weeks. Several factors may explain this finding. First, the BBS comprises 14 items covering multiple dimensions of balance, including weight shifting, static balance, and dynamic balance (31); this multidimensionality may contribute to substantial inter-study variability, resulting in insufficient statistical power for subgroup analyses. Second, BFRT protocols varied considerably across studies, with substantial differences in compression site (proximal thigh vs. mid-femur), pressure levels (70–250 mmHg), and training modalities (combined with resistance, vibration, or cycling). Third, improvements in balance function may occur early and plateau after a certain intervention duration, diminishing the observable differences between time-based subgroups. Notably, the >4-week subgroup demonstrated a clinically meaningful improvement trend (MD = 5.02, *p* < 0.00001), and the overall effect remained significant, confirming that BFRT effectively enhances balance function. The underlying mechanisms may involve increased strength in proximal lower limb muscles (e.g., gluteals, quadriceps), enhanced proprioceptive input, and optimized postural control strategies.

Regarding walking capacity, BFRT significantly improved 6MWT performance (MD = 9.73, *p* < 0.00001) but showed no significant improvement on the TUGT (MD = 3.99, *p* = 0.19). This differential effect suggests that BFRT influences distinct dimensions of walking function through different mechanisms. The 6MWT primarily reflects walking endurance. Its improvement likely stems from BFRT-induced enhancements in muscle metabolic efficiency, oxidative enzyme activity, and capillary density. Specifically, BFRT promotes a shift in slow-twitch muscle fibers toward oxidative metabolism during low-intensity exercise, delaying fatigue onset and enabling patients to cover greater distances within 6 min (32). In contrast, the TUGT emphasizes gait agility and complex motor sequencing. It assesses the total time required to rise from a seated position, walk 3 meters, turn, and return to sitting. This test relies more heavily on lower limb muscle power, core control, coordination, and movement transition agility (33). While BFRT effectively enhances muscle strength and cross-sectional area, its impact on neural drive frequency, motor unit synchronization, and joint coordination patterns—key determinants of TUGT performance—may be limited. Furthermore, the TUGT involves complex movements such as turning and sit-to-stand transitions, which require higher-level central integration and motor planning. As an intervention primarily relying on local metabolic stimulation, BFRT may have limited effects on improving these central motor control abilities (34). Therefore, to comprehensively enhance walking ability—particularly agility and coordination—future rehabilitation protocols should integrate task-oriented training, balance training, and core muscle training with BFRT, forming a multidimensional rehabilitation approach.

From a physiological mechanism perspective, BFRT induces localized ischemia–reperfusion effects through compression-decompression cycles, activating the mTOR signaling pathway, promoting anabolic hormone secretion (e.g., growth hormone and IGF-1), and increasing muscle protein synthesis, ultimately leading to muscle fiber hypertrophy and strength gains (35, 36). Simultaneously, the accumulation of metabolic byproducts (e.g., lactate, hydrogen ions) stimulates group III/IV afferent nerves, heightens motor cortex excitability, and promotes neuromuscular functional remodeling (37). These mechanisms predominantly exhibit cumulative effects, requiring repeated stimulation and sufficient duration to translate into significant functional gains. This biological rationale further supports the observation that intervention duration >4 weeks was necessary to achieve significant improvements in multiple outcomes in the present study.

The clinical significance of the functional improvements observed in this study must be interpreted in conjunction with the minimum clinically important difference (MCID) thresholds. For the BBS, the MCID in stroke patients is 5–6 points (38, 39). In this study, the pooled effect size from the primary analysis was 4.52 points, which approached this threshold; after excluding high-risk studies, the effect size was 5.31 points, meeting the MCID threshold. However, the latter was no longer statistically significant (*p* = 0.09), likely due to the reduced sample size and consequent loss of statistical power. The MCID for the FMA-LE is 4–5 points (40); the pooled effect size for interventions lasting >4 weeks was 4.38 points, meeting this threshold. The MCID for the 6MWT is 30–50 meters (41); the 9.73-meter improvement observed in this study did not reach this threshold. The MCID for the MBI is 10–15 points (42); the effect size for interventions lasting >4 weeks was 10.50 points, approaching this threshold. From the perspective of functional impact, improvements in the BBS are closely associated with reduced fall risk (43), while enhancements in lower-limb motor function and activities of daily living directly promote patients’ independent walking ability and quality of life. Collectively, these findings suggest that blood flow restriction training has potential clinical value in improving balance, lower-limb motor function, and activities of daily living; however, its effect on walking endurance appears limited.

This study has several limitations. First, the number of included studies and sample sizes were limited, with fewer studies available for certain outcome measures (e.g., only three studies for the 6MWT, potentially affecting statistical power. Second, BFRT protocols varied significantly across studies, with substantial differences in compression site, pressure level, cuff type, intervention duration, and frequency, which may have contributed to clinical heterogeneity. Third, constrained by the original study designs, most trials did not implement blinding of patients or intervention personnel. Although most outcomes were objective measures, implementation and measurement biases cannot be entirely ruled out. Fourth, this study did not perform subgroup analyses based on factors such as sex, age, stroke type, or time since stroke, which may have introduced heterogeneity. Fifth, sensitivity analysis revealed that after excluding the two high-risk studies, the pooled effect for the BBS was no longer statistically significant [MD = 5.31, 95% CI (−0.82, 11.44), *p* = 0.09], suggesting that the results of the primary analysis may have been influenced by low-quality studies. Thus, the robustness of the findings requires further validation. Future research requires large-scale, high-quality multicenter RCTs to thoroughly investigate optimal BFRT protocols and intervention parameters.

## 5 Conclusion

In summary, blood flow restriction training represents an effective therapeutic approach for patients with lower limb dysfunction after stroke, significantly improving balance and walking endurance. Subgroup analyses indicate that interventions lasting longer than 4 weeks can further enhance lower limb motor function and activities of daily living, with more pronounced improvements in lower limb motor function observed in patients during the subacute phase. Therefore, it is recommended that blood flow restriction training be incorporated into routine rehabilitation programs in clinical practice, with particular attention to the potential benefits of long-term interventions (>4 weeks) and early intervention (subacute phase). However, given the limitations of the current included studies—including sample size, variability in intervention protocols, and methodological quality—the above conclusions warrant further validation through additional large-scale, high-quality studies.

## Data Availability

The original contributions presented in the study are included in the article/supplementary material, further inquiries can be directed to the corresponding authors.

## References

[1] Feigin VL Owolabi MO. World stroke organization–lancet neurology commission stroke collaboration group. Pragmatic solutions to reduce the global burden of stroke: a world stroke organization-lancet neurology commission. Lancet Neurol. (2023) 22:1160–206. doi: 10.1016/S1474-4422(09)70025-0 37827183 PMC10715732

[2] Feigin VL Brainin M Norrving B Martins SO Pandian J Lindsay P . World stroke organization: global stroke fact sheet 2025. Int J Stroke. (2025) 20:132–44. doi: 10.1177/17474930241308142, 39635884 PMC11786524

[3] Li J Zhong D Ye J He M Liu X Zheng H . Rehabilitation for balance impairment in patients after stroke:a protocol of a systematic review and network meta-analysis. BMJ Open. (2019) 9:e026844. doi: 10.1136/bmjopen-2018-026844, 31326927 PMC6661695

[4] Miyata K Hasegawa S Iwamoto H Otani T Kaizu Y Shinohara T . Comparing the measurement properties and relationship to gait speed recovery of the Mini- balance evaluation systems test and the Berg balance scale in ambulatory individuals with subacute stroke. Phys Ther Res. (2020) 23:72–8. doi: 10.1298/ptr.E10004, 32850282 PMC7344358

[5] Moore SA Boyne P Fulk G Verheyden G Fini NA. Walk the talk: current evidence for walking recovery after stroke, future pathways and a Mission for research and clinical practice. Stroke. (2022) 53:3494–505. doi: 10.1161/STROKEAHA.122.038956, 36069185 PMC9613533

[6] Wu S Wu B Liu M China Stroke Study Collaboration. Stroke in China: advances and challenges in epidemiology, prevention, and management. Lancet Neurol. (2019) 18:394–405. doi: 10.1016/S1474-4422(18)30500-3, 30878104

[7] Watson R Sullivan B Stone A Jacobs C Malone T Heebner N . Blood flow restriction therapy: an evidence-based approach to postoperative rehabilitation. JBJS Rev. (2022) 10. doi: 10.2106/JBJS.RVW.22.00062 36191086

[8] Lorenz DS Bailey L Wilk KE Mangine RE Head P Grindstaff TL . Blood flow restriction training. J Athl Train. (2021) 56:937–44. doi: 10.4085/418-20, 34530434 PMC8448465

[9] Hughes L Paton B Rosenblatt B Gissane C Patterson SD. Blood flow restriction training in clinical musculoskeletal rehabilitation: a systematic review and meta-analysis. Br J Sports Med. (2017) 51:1003–11. doi: 10.1136/bjsports-2016-097071, 28259850

[10] Wortman RJ Brown SM Savage-Elliott I Finley ZJ Mulcahey MK. Blood flow restriction training for athletes: a systematic review. Am J Sports Med. (2021) 49:1938–44. doi: 10.1177/0363546520964454 33196300

[11] Ning Y Sheng T Yanlan M. Progress in the application of blood flow restriction training in muscle exercise for patients. Chin J Rehabilit Med. (2022) 37:540–5. doi: 10.3969/j.issn.1001-1242.2022.04.020

[12] Kjeldsen SS Næss-Schmidt ET Lee M de Oliveira CQ Nielsen JF Stubbs PW. Blood flow restriction exercise of the tibialis anterior in people with stroke: a preliminary study. J Integr Neurosci. (2022) 21:53. doi: 10.31083/j.jin2102053, 35364641

[13] Xiaodie T Shujuan Y. Progress of blood flow restriction training in rehabilitation medicine. China Sports Sci Technol. (2023) 59:81–5. doi: 10.16470/j.csst.2021134

[14] Cummings M Madhavan S. Blood flow modulation to improve motor and neurophysiological outcomes in individuals with stroke: a scoping review. Exp Brain Res. (2024) 242:2665–76. doi: 10.1007/s00221-024-06941-5, 39368025

[15] Feng G. Chinese expert consensus on endovascular treatment of acute ischemic stroke. Chin J Cerebrovasc Dis. (2014) 11:556–60. doi: 10.3969/j.issn.1672-5921.2014.010.012

[16] Cumpston M Li T Page MJ Chandler J Welch VA Higgins JP . Updated guidance for trusted systematic reviews: a new edition of the Cochrane handbook for systematic reviews of interventions. Cohrane Database Syst Rev. (2019):10. doi: 10.1002/14651858.ED000142 PMC10284251 31643080

[17] Yang B. Effect of blood flow restriction training method on muscle and limb function rehabilitation in obese stroke hemiplegic patients. Liaoning Med J. (2021) 35:98–100.

[18] Yan D Xuemei C Li L. Effects of blood flow restriction combined with resistance training on lower limb function in elderly stroke patients with hemiplegia. Jilin Med Sci. (2022) 43:800–2. doi: 10.3969/j.issn.1004-0412.2022.03.089

[19] Jinnan DU Baozhen GUO Qunyou WANG. Evaluation of the efficacy of blood flow restriction training combined with repetitive transcranial stimulation on the effect of lower limb function after stroke. Chin Foreign Med. (2022) 41:23–26+35. doi: 10.16662/j.cnki.1674-0742.2022.12.023

[20] Yali F Fanglin W Yi Z Wenwen Y Zhimin X Ying Y . Effects of blood flow restriction combined with exercise training on lower limb function and walking ability in stroke patients. Chin J Rehabilit Med. (2023) 38:331–6. doi: 10.3969/j.issn.1001-1242.2023.03.008

[21] Wenjing X Xingguo Z Ting Z Bo L Wenli C Zhen S . Effects of blood flow restriction combined with low-intensity resistance exercise on lower limb function and surface EMG in stroke patients. Chin J Rehabilit Med. (2023) 38:46–51. doi: 10.3969/j.issn.1001-1242.2023.01.008

[22] Liangwen S Chunxia W Miao L Min L Shaojun G Bo W . Effects of whole-body vibration combined with blood flow restriction training on motor function and community activity ability of elderly stroke patients with hemiplegia. J Pract Med. (2024) 40:2874–9. doi: 10.3969/j.issn.1006-5725.2024.20.010

[23] Ahmed I Mustafaoglu R Erhan B. The effects of low-intensity resistance training with blood flow restriction versus traditional resistance exercise on lower extremity muscle strength and motor functionin ischemic stroke survivors: a randomized controlled trial. Top Stroke Rehabil. (2024) 31:418–29. doi: 10.1080/10749357.2023.2259170, 37724785

[24] Jianting G Renhua L Qing L Jianwei J Lu Z Yan Z . Clinical study of blood flow restriction training combined with head acupuncture in the treatment of balance function in stroke patients. J Hunan Univ Tradit Chin Med. (2025) 45:480–5. doi: 10.3969/j.issn.1674-070X.2025.03.013

[25] Xiaodie T Shujuan Y Liang F. Observation on the efficacy of blood flow restriction training on balance function of ischemic stroke patients. Shanxi Med J. (2025) 54:900–4. doi: 10.3969/j.issn.0253-9926.2025.12.004

[26] Jian S Yaorui G Li B Shouli F Lihong J Zheng W . Effects of blood flow restriction combined with suspension training on lower limb function and walking ability in stroke patients. China. Rehabilitation. (2025) 40:11–5. doi: 10.3870/zgkf.2025.01.002

[27] Feng Y Wen F Ahmad I Chen Y Ye W Jiang H . Does exercise training combined with blood flow restriction improve muscle mass, lower extremity function, and walking capacity in hemiplegic patients? A randomized clinical trial. Top Stroke Rehabil. (2025) 32:800–9. doi: 10.1080/10749357.2025.2482390, 40159947

[28] Wu L Xu G Wu Q. The effect of the Lokomat® robotic-orthosis system on lower extremity rehabilitation in patients with stroke: a systematic review and meta-analysis. Front Neurol. (2023) 14:1260652. doi: 10.3389/fneur.2023.1260652, 38125828 PMC10730677

[29] Downs S Marquez J Chiarelli P. The Berg balance scale has high intra- and inter-rater reliability but absolute reliability varies across the scale: a systematic review. J Physiother. (2013) 59:93–9. doi: 10.1016/S1836-9553(13)70161-9, 23663794

[30] Stinear CM Lang CE Zeiler S Byblow WD. Advances and challenges in stroke rehabilitation. Lancet Neurol. (2020) 19:348–60. doi: 10.1016/S1474-4422(19)30415-6 32004440

[31] Blum L Korner-Bitensky N. Usefulness of the Berg balance scale in stroke rehabilitation: a systematic review. Phys Ther. (2008) 88:559–66. doi: 10.2522/ptj.20070205, 18292215

[32] Agarwala P Salzman SH. Six-minute walk test: clinical role, technique, coding, and reimbursement. Chest. (2020) 157:603–11. doi: 10.1016/j.chest.2019.10.014, 31689414 PMC7609960

[33] Yoo JE Jang W Shin DW Jeong SM Jung HW Youn J . Timed up and go test and the risk of Parkinson's disease: a nation-wide retrospective cohort study. Mov Disord. (2020) 35:1263–7. doi: 10.1002/mds.28055, 32293759

[34] Erickson LN Lucas KCH Davis KA Jacobs CA Thompson KL Hardy PA . Effect of blood flow restriction training on quadriceps muscle strength, morphology, physiology, and knee biomechanics before and after anterior cruciate ligament reconstruction: protocol for a randomized clinical trial. Phys Ther. (2019) 99:1010–9. doi: 10.1093/ptj/pzz062, 30951598 PMC6665950

[35] Hwang PS Willoughby DS. Mechanisms behind blood flow-restricted training and its effect toward muscle growth. J Strength Cond Res. (2019) 33:S167–79. doi: 10.1519/JSC.0000000000002384, 30011262

[36] Dankel SJ Jessee MB Abe T Loenneke JP. The effects of blood flow restriction on upper-body musculature located distal and proximal to applied pressure. Sports Med. (2016) 46:23–33. doi: 10.1007/s40279-015-0407-7, 26446893

[37] Bobes AC Issa-Khozouz SP Fernández-Matías R Pecos-Martín D Achalandabaso-Ochoa A Fernández-Carnero S . Comparison of blood flow restriction training versus non-occlusive training in patients with anterior cruciate ligament reconstruction or knee osteoarthritis: a systematic review. J Clin Med. (2020) 10:68. doi: 10.3390/jcm10010068, 33375515 PMC7796201

[38] Hiengkaew V Jitaree K Chaiyawat P. Minimal detectable changes of the Berg balance scale, Fugl-Meyer assessment scale, timed "up & go" test, gait speeds, and 2-minute walk test in individuals with chronic stroke with different degrees of ankle plantarflexor tone. Arch Phys Med Rehabil. (2012) 93:1201–8. doi: 10.1016/j.apmr.2012.01.014, 22502805

[39] Donoghue D Stokes EK. How much change is true change? The minimum detectable change of the Berg balance scale in elderly people. J Rehabil Med. (2009) 41:343–6. doi: 10.2340/16501977-0337, 19363567

[40] Pandian S Arya KN Kumar D. Minimal clinically important difference of the lower-extremity fugl-meyer assessment in chronic-stroke. Top Stroke Rehabil. (2016) 23:233–9. doi: 10.1179/1945511915Y.0000000003, 27086865

[41] Tang A Eng JJ Rand D. Relationship between perceived and measured changes in walking after stroke. J Neurol Phys Ther. (2012) 36:115–21. doi: 10.1097/NPT.0b013e318262dbd0, 22850336 PMC3501529

[42] Hsieh YW Wang CH Wu SC Chen PC Sheu CF Hsieh CL. Establishing the minimal clinically important difference of the Barthel index in stroke patients. Neurorehabil Neural Repair. (2007) 21:233–8. doi: 10.1177/1545968306294729, 17351082

[43] Muir SW Berg K Chesworth B Speechley M. Use of the Berg balance scale for predicting multiple falls in community-dwelling elderly people: a prospective study. Phys Ther. (2008) 88:449–59. doi: 10.2522/ptj.20070251, 18218822

